# Processes Underpinning Successful Co‐Design: Lessons From a Digital Health Project

**DOI:** 10.1111/hex.70272

**Published:** 2025-05-22

**Authors:** Catherine Burns, Monique F. Kilkenny, Tara Purvis, Seana L. Gall, Christine Farmer, Seamus Barker, Brenda Booth, Janet E. Bray, Dominique A. Cadilhac, Jan Cameron, Lachlan L. Dalli, Stephanie Ho, Eleanor Horton, Timothy Kleinig, Lisa Murphy, Mark R. Nelson, Muideen T. Olaiya, Amanda G. Thrift, Rosanne Freak‐Poli

**Affiliations:** ^1^ Stroke and Ageing Research, Department of Medicine School of Clinical Sciences at Monash Health Monash University Clayton Australia; ^2^ Stroke Theme, Florey Institute of Neuroscience and Mental Health University of Melbourne Heidelberg Australia; ^3^ Menzies Institute for Medical Research University of Tasmania Hobart Australia; ^4^ Stroke Foundation Melbourne Australia; ^5^ School of Public Health and Preventive Medicine Monash University Melbourne Australia; ^6^ Department of Neurology Royal Adelaide Hospital Adelaide Australia; ^7^ Department of Medicine University of Adelaide Adelaide Australia

**Keywords:** co‐design, consumer engagement, digital health, focus groups

## Abstract

**Background:**

Co‐design helps to align research with end‐user needs, but there is no consistent method for reporting co‐design methodology and evaluation. We share our experiences co‐designing the Love Your Brain digital platform for stroke prevention. We evaluated the core attributes that guided our co‐design approach, including recruitment, focus group coordination, participant engagement and satisfaction.

**Methods:**

Online co‐design focus groups were conducted fortnightly (May 2023 to March 2024) with two cohorts (health knowledge experts and community members; *n* = 8 sessions per cohort) to design the content and structure of Love Your Brain. Snowballing methods and purposive sampling were used to recruit participants in Australia. Coordination involved tracking the time spent by the research team for one round of focus groups. Participant engagement was measured through focus group attendance and survey feedback and analysed using descriptive statistics and thematic analysis. Count and length of verbal and/or written contributions during focus groups were summarised with descriptive statistics as a measure of engagement, with differences between cohorts assessed using *χ* ^2^/Wilcoxon rank‐sum tests. Participant satisfaction was evaluated using survey responses and input at a final evaluative focus group.

**Results:**

Sixteen health knowledge experts (clinicians/researchers) and 28 community members expressed interest, of which 10 health knowledge experts and 12 community members (including 9 people with lived experience of stroke) participated. Conducting two identical focus groups required 29 h of project manager/coordinator time, 8–11 h for facilitators and 6.5–8.5 h for chief investigators. Most participants (86%) attended ≥ 5/8 focus groups. Engagement was enhanced through pre‐reading material, structured/well‐organised focus groups and experienced facilitators. All participants contributed at each focus group, with varying levels of input. Health knowledge experts preferred written contributions over verbal contributions and wrote longer messages compared to community members. Community members spoke for a longer duration than health knowledge experts. Participant satisfaction was high, with participants reporting that the research team ‘always valued our opinions’. Importance was placed on a final evaluative focus group, and participants stated that their contributions were incorporated into the final product.

**Conclusion:**

Our research emphasises relationships between coordination, participant engagement and satisfaction and the importance of considering resourcing requirements for successful co‐design of digital health projects.

**Patient or Public Contribution:**

People with lived experience, including caregivers of people with stroke, and members of the public, participated in the co‐design focus groups. The Love Your Brain Management Committee includes people with lived experience of stroke who contributed to the oversight of this study and the preparation of this manuscript.

## 1 Introduction

Co‐design in health research is a collaborative approach in which researchers and end‐users of research actively partner throughout the research process [1, 2]. End‐users of research may include a variety of different stakeholders, such as patients, clinical and non‐clinical staff, community members and policymakers. Co‐design is a powerful tool to align research aims and the design of interventions with community needs, by creating an opportunity for knowledge exchange, and shared power, trust and decision‐making among stakeholders [3]. This can result in a sense of empowerment and accomplishment for those involved [4, 5]. Specifically for digital health interventions, co‐design is essential to optimise success by ensuring the needs of users are adequately incorporated [6, 7]. Active stakeholder engagement bridges the gap between research and practice [8], to enhance the relevance, usefulness and acceptability of health interventions [9].

There is no consistent way to report co‐design methods [10]. Despite the existence of co‐design theoretical frameworks, all components are rarely included, and reporting is often limited to the quantity and quality of outputs produced [11, 12]. The processes of co‐design include the project management strategies selected by the research team, composition of the group, tools and activities used during co‐design sessions, decision‐making processes, engagement of participants, and the financial investment related to participant reimbursement and staff time [12, 13]. Evaluation of the person‐level experience of co‐design can include attributes such as satisfaction, value‐proposition, achievement of a positive outcome or impact, and sense of equality and respect throughout the process [11, 13].

We aimed to describe our experiences and knowledge gained from co‐designing a digital platform for stroke prevention called Love Your Brain. We examined the attributes and people that underpinned our co‐design processes, in terms of recruitment, coordination of focus groups (including time and resources), participant engagement and satisfaction. Here, we share our methodology and evaluation to benefit other researchers planning to use co‐design for digital health interventions.

## 2 Materials and Methods

### 2.1 Research Context

Love Your Brain is a 3‐year research project with the goal of developing and evaluating a digital platform for the primary prevention of stroke in Australia. Co‐design is integral to Love Your Brain, with consumers and people with lived experience working in partnership with researchers to guide the entire project. This project leverages the success of the Stroke Foundation StrokeSafe program, which provides community‐based education for primary stroke prevention. StrokeSafe presentations are effective in improving short‐term knowledge of stroke; however, this knowledge diminishes over time without reinforcement [14]. The Love Your Brain digital platform will include a Massive Open Online Course (MOOC) and text messaging system to deliver stroke prevention information over 12 weeks. The aims of Love Your Brain are to increase visits to medical practitioners for assessment of stroke risk factors, improve knowledge retention of stroke risk factors and increase healthy behaviours (e.g., adherence to prescribed medication).

We report on the attributes that underpinned the co‐design process of the digital platform. In a separate report, we have outlined how the needs and preferences of participants involved in the co‐design process were used to develop the digital platform [15].

### 2.2 Study Design

We undertook a distributed co‐design methodology where stakeholder groups were engaged separately. This design intends to mitigate the inherent power hierarchy in healthcare between people with expertise and community members to give each cohort an equal voice and foster cohesion [16, 17]. We evaluated our co‐design processes using a mixed‐methods approach. We have been guided by aspects of the Co‐Design Evaluation Framework, which was developed to assist researchers in evaluating their co‐design processes and impacts [11]. This framework includes components that underpin co‐design, including the processes and people, as well as the intended beneficiaries beyond those involved in the co‐design (e.g., the wider community) [11]. We applied this framework retrospectively to identify areas of success and improvement in our co‐design process, including research processes (coordination), group processes (recruitment and participant engagement) and people (participant satisfaction).

### 2.3 Study Population

Two separate cohorts of up to 15 stakeholders each participated in the co‐design focus groups. To allow for diverse perspectives and maximal engagement, we aimed to have 8–12 participants attend each focus group [18]. One cohort comprised health knowledge experts, defined as professionals involved in stroke health education or health promotion (e.g., clinicians and researchers) with ≥ 1 year of experience. The other cohort comprised community members, with or without a lived experience of stroke. The inclusion criteria for both groups included being aged ≥ 18 years, proficiency in the English language, able to access the internet and living in the community.

### 2.4 Recruitment and Sampling

Investigators circulated an email containing the recruitment flyer to their professional contacts, existing clinical and research networks, community reference groups, consumer councils and newsletters (Australian Stroke Clinical Registry and Stroke Foundation) from 18 April to 9 May 2023 (Supporting Table I). Potential participants completed an expression of interest form in Qualtrics XM (Qualtrics, Utah) that included demographic information (Supporting Table II).

**Figure 1:**
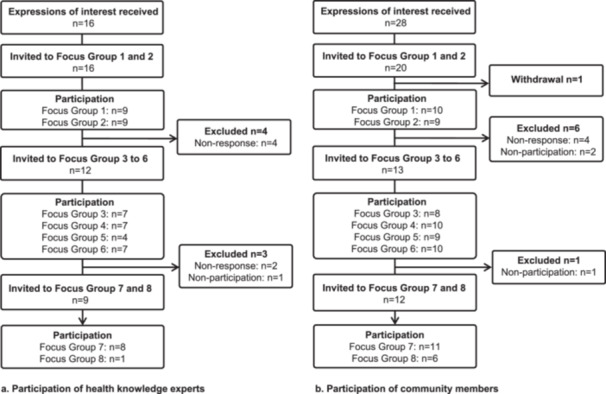
Recruitment and participation in focus groups.

Purposive sampling was used to ensure diversity of participants based on age, gender, past history of stroke, geographical location, level of education (community members) and profession (health knowledge experts). After the first two focus groups, invitations were only sent to participants who had demonstrated engagement through prior attendance or response to invitations (Figure 1).

### 2.5 Focus Group Coordination

**Figure 2:**
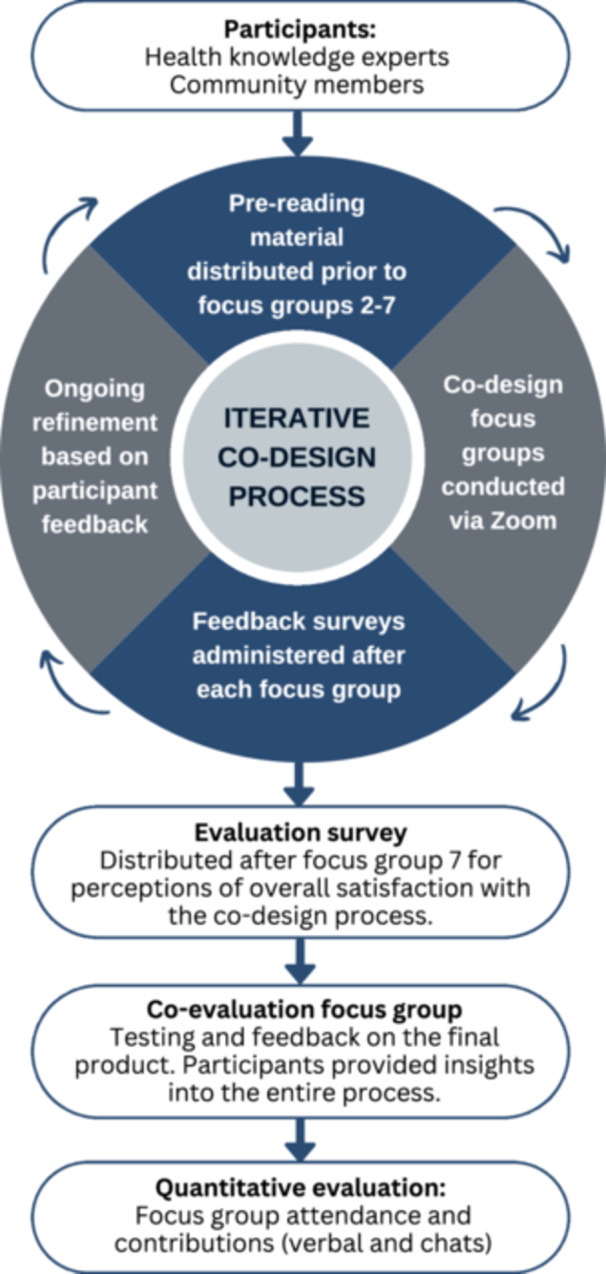
The iterative co‐design process and evaluation methods.

We planned eight semi‐structured focus groups per cohort over 6 months. An iterative approach was used, whereby discussions during focus groups and feedback from surveys facilitated refinement of the co‐design process and content (Figure 2). Seven focus groups were allocated for the pre‐design and generative phases of co‐design, and the eighth focus group was for the evaluative phase [19]. In the pre‐design phase, we explored participants' thoughts and experiences, and then the generative phase focused on new ideas for the function, content and design of the digital platform [4]. Participants shared their ideas and experiences and were active partners in this research. The insights gained from the initial seven focus groups per cohort were used to inform and develop the digital platform over 7 months. The evaluative phase allowed participants to test the digital platform for 2 weeks and then reconvene for a final focus group to share their feedback on the output (Figure 2). At the final focus group, there was also a discussion about the overall experience of co‐design.

Focus groups were conducted via Zoom video conferencing software (Zoom Video Communications, California) and were up to 60 min in duration. Each fortnight, the health knowledge expert focus group occurred on Tuesday at 1 pm, followed by the community member focus group on Thursday at 1 pm. Focus groups were held on the same day and time throughout, with the exception of the final focus groups, which were altered due to facilitator availability. The day and time for focus groups were chosen to accommodate the availability of the research team, with consideration of different time zones across Australia, and the likely availability of working professionals at lunchtime.

Focus groups were facilitated by the same experienced qualitative researchers with a background in stroke research and clinical care (health knowledge experts: T.P., female, PhD; community members: S.B., male, PhD). Each focus group started with an introduction to the project and a summary of the previous focus group discussion, followed by a semi‐structured discussion. The focus groups incorporated activities such as viewing sample videos and images curated by the research team, brainstorming and facilitated discussion using prompt questions. The topics discussed in the focus groups comprised the most important content to include, type and depth of information, formatting and appearance, and functionality of each component (e.g., text message frequency) of the digital platform. Throughout the co‐design process, discussions with each cohort iteratively informed the key considerations for the digital platform and guided the topics and content for subsequent focus groups. Additional research staff (M.F.K., S.L.G., C.F., C.B. and R.F.) supported the facilitator to stimulate engagement and participation by annotating a virtual whiteboard with participant responses, launching polls within Zoom, monitoring the chat function, providing technical support and responding to participant queries as required. To support repeat attendance, reminder emails containing the Zoom link were sent to participants 1 week before and 1 day before each focus group. For simplicity, the same Zoom link was used for all focus groups. After each focus group, the facilitator advertised the next focus group date and time, and the project coordinator circulated an email with a link to provide feedback on the current focus group, with instructions for reimbursement (supermarket gift voucher or bank transfer). Reimbursement for participants' time and contribution was in line with the Monash Partners Remuneration and Reimbursement Guidelines [20]. Gift vouchers were distributed after each focus group. Bank transfers for the total reimbursement amount were processed after the final focus groups. All participants received a certificate of participation at the end of the co‐design process.

### 2.6 Data Collection

#### 2.6.1 Recruitment

Correspondence with participants (expressions of interest, focus group invitations, participant responses and withdrawal) was tracked in Qualtrics XM (Qualtrics, Utah) and an Excel spreadsheet (Microsoft Corporation, Washington).

#### 2.6.2 Focus Group Coordination

To estimate research staff time required to prepare, coordinate and facilitate the focus groups, a time tracking record was kept for one indicative fortnight (12–25 June 2023). This period included two identical focus groups (one per cohort) and a project management committee meeting. We chose this period as it was in the middle of the co‐design process after we had successfully developed and implemented systems for planning, implementing and evaluating the focus groups. Research staff time was recorded for the project manager, project coordinator, two chief co‐investigators and two focus group facilitators.

#### 2.6.3 Participant Engagement

Following verbal consent from participants, the sessions were audio and video recorded via Zoom, including automated transcription. We collected details of all chat messages, poll responses, virtual whiteboard notes, researcher notes and attendance reports. We developed a novel measure of participant engagement for verbal contributions (using a count and duration in seconds) and written contributions (count and character length). These metrics were captured for the first seven focus groups for each cohort and recorded in a REDCap database hosted at Monash University [21, 22]. Survey response rate and pre‐reading video view counts were also used to measure participant engagement.

#### 2.6.4 Participant Satisfaction

After each of the first six focus groups, participants could complete a post‐session feedback survey using Qualtrics XM (Qualtrics, Utah; Supporting Table III). This survey comprised questions on group structure, functionality and administration using a mixture of open and closed questions, including Likert scales. Free text fields were included for participants to provide additional responses to the focus group prompt questions.

After the seventh focus group, participants were sent a final evaluation survey to ascertain their feedback on the co‐design process. The final evaluation survey comprised 10 closed questions on Likert scales and six short answer open‐ended questions to provide more in‐depth responses about their experiences, process and outcomes of participating in the focus groups (Supporting Table IV). Feedback related to the co‐design process was also collected verbally and via the chat at the final evaluation focus group.

### 2.7 Statistical Analysis

Descriptive statistics were used to summarise the characteristics of participants and engagement metrics. Engagement metrics were compared between cohorts using *χ* ^2^ tests for categorical variables and Wilcoxon rank‐sum tests for non‐parametric continuous variables. A two‐sided *p* value < 0.05 was considered statistically significant. Data were analysed using Stata/SE version 18 (StataCorp, Texas). To our knowledge, there is no validated method to measure participant engagement in focus groups, so evaluation metrics were chosen in line with the co‐design objectives. Quantitative data from the final evaluative focus group for each cohort were not included in the participant engagement data.

One author (C.B.) summarised and thematically analysed [23] free‐text data from all surveys and feedback provided at the final evaluative focus groups. Ongoing discussions between the research team responsible for the analysis ensured that data were being interpreted and summarised appropriately to reflect the intended meaning.

### 2.8 Ethics

Ethical approval for this study was received from the Monash University Human Research Ethics Committee (#35899). Universal Trial Number U1111‐1305‐2964. Participation in the co‐design focus groups was voluntary. Consent was obtained before and at the beginning of each focus group, including consent to record.

## 3 Results

### 3.1 Recruitment and Participants

A total of 44 expressions of interest were received (16 health knowledge experts and 28 community members; Figure 1). Five additional enquiries that were received from health knowledge experts after the closing date were not considered.

All health knowledge experts (*n* = 16) were invited to participate in the first two focus groups. Twenty of 28 eligible community members were blindly selected (participant name not visible), allowing for diversity of location, gender, lived experience of stroke and age category. Of the 16 health knowledge experts invited, 10 attended at least one focus group. Of the 20 community members invited, 12 attended at least one focus group.

#### 3.1.1 Characteristic of Health Knowledge Experts

Of the 10 health knowledge expert participants, the majority were aged ≥ 35 years (60%), female (80%) and from either the states of New South Wales (60%) or Victoria (40%; Supporting Table V). All participants were university educated, with 80% holding postgraduate qualifications. Half of this cohort were clinicians (speech pathologist, occupational therapist, physiotherapist and nurse), and half were researchers, with six (60%) having ≥ 10 years' experience in their profession.

#### 3.1.2 Characteristic of Community Members

Over half of the 12 community member participants were aged < 65 years (58%) and female (58%; Supporting Table V). There was representation from six states and territories in Australia, and 92% (*n* = 11) identified as Australian. All 12 community members were Stroke Foundation StrokeSafe program speakers and had completed higher education, with nine (75%) attaining a Bachelor's Degree or higher. Nine (75%) participants had previously experienced stroke or transient ischaemic attack, two (17%) participants were currently caring for someone who had experienced a stroke and one (8%) participant had no personal experience with stroke. Two participants (17%) reported no risk factors for cardiovascular disease.

### 3.2 Focus Group Coordination

The first seven focus groups dedicated to the pre‐design and generative phases were conducted over a 14‐week period (May–August 2023), followed by the final evaluative focus group in March 2024.

To prepare for each focus group, research staff (*n* = 6, including the project manager, project coordinator, two chief co‐investigators and two focus group facilitators) participated in at least two meetings to outline the anticipated content and delivery then attended the focus groups for both cohorts. The project manager and project coordinator also spent time writing summaries of the focus group discussions, preparing pre‐reading and content for each focus group, scheduling invitations, corresponding with participants and arranging reimbursement. It was estimated that the delivery of two identical 60‐min focus groups required 29 h of project team time (16 h project manager and 13 h project coordinator), 8–11 h for each focus group facilitator and 6.5–8.5 h for the project‐coordinating chief investigators during the fortnight. In addition, the project management committee had a 1‐h meeting every 2 months to provide oversight, generally with 10 investigators and 5 project team members in attendance. Indirect costs associated with facilitating the focus groups included software licences for video conferencing, video editing and survey distribution.

Reimbursement was offered to all participants who attended focus groups, but not all participants chose to be reimbursed. Across all focus groups, the total amount of reimbursement budgeted was AU$5000, with an actual cost of AU$4400. Due to the complexity of internal processes for reimbursement via bank transfer, many participants opted to receive gift vouchers. All respondents (*n* = 15) to the final evaluation survey agreed that they were fairly reimbursed.

### 3.3 Participant Engagement

#### 3.3.1 Response to Post‐Session Feedback Survey and Final Evaluation Survey

The majority of participants responded to post‐session feedback surveys following the first two focus groups (response rate: 89% and 67% health knowledge experts and 80% and 78% community members). There were no additional post‐session feedback survey responses from the health knowledge experts for the remaining focus groups. Six responses were received from community members after focus groups 4, 5 or 6. The final evaluation survey was distributed to 21 participants (9 health knowledge experts and 12 community members) with a response rate of 71% (67% health knowledge experts, *n* = 6; 75% community members, *n* = 9).

#### 3.3.2 Attendance

Nineteen (86%) participants attended five or more focus groups for their cohort (8 health knowledge experts and 11 community members), with four attending all seven pre‐design and generative co‐design focus groups for their cohort (two health knowledge experts and two community members). Responses from the post‐session feedback survey following the first focus group indicated that the chosen day and time suited most respondents (*n* = 15, 94%). However, these responses were from participants who were available to attend the first focus group.

Suggestions to improve future participation from health knowledge experts included the option for face‐to‐face discussion and scheduling focus groups outside work hours. One respondent commented that it was ‘difficult to commit to so many sessions’, and another suggested notifying participants of all focus group dates and times at the start would be beneficial. In contrast, one community member commented that they were ‘always pleased to participate in any way [they] can which may assist stroke prevention’, with two participants noting that ‘nothing’ was needed to improve participation in future.

#### 3.3.3 Pre‐Reading Material

During the first focus group, it was established that pre‐reading distributed before each focus group would be useful preparation (Figure 2). For the remaining focus groups, participants were emailed a 1‐ to 2‐page document outlining the topic for discussion, to read at their discretion, including links to relevant videos created by the research team (Figure S1). The median views per video was 16 (interquartile range 16–19), indicating that approximately 68% of participants across both cohorts watched the pre‐reading videos (or fewer with repeat views). In the final evaluation survey, the majority of both cohorts agreed or strongly agreed that the pre‐reading was useful and engaging (*n* = 4 [66%] health knowledge experts and *n* = 9 [100%] community members). Two community members commented that the pre‐reading gave them the ability to think ahead of time, thereby supporting their input to the discussion. In addition, three written responses to the pre‐reading prompt questions were received from the community member cohort ahead of the third (*n* = 1) and fifth (*n* = 2) focus groups.

#### 3.3.4 Co‐Design Processes

**Figure 3:**
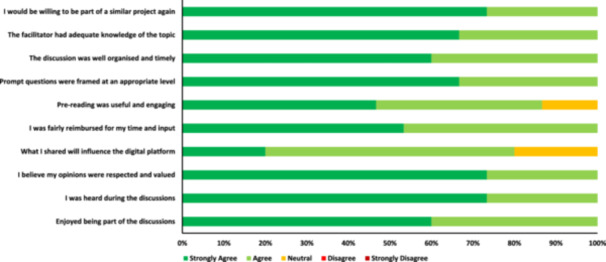
Evaluation of the co‐design process (*n* = 15 responses; 6 responses from health knowledge experts and 9 responses from community members).

All respondents (*n* = 15) to the final evaluation survey from both cohorts agreed that the focused prompt questions used to stimulate discussion during the focus groups were framed at an appropriate level, the discussion was well organised and timely, and the facilitator had adequate knowledge of the topic (Figure 3). Health knowledge experts commented that the co‐design process was ‘very well facilitated and structured’, and a participant with previous co‐design experience commented that they ‘learnt the value of having structure around co‐design discussions’. The community members agreed, with one participant stating that there was ‘a true understanding of co‐design’.

Participants from both cohorts commented that ‘all activities were well facilitated’ and the focus groups were ‘very interesting and engaging’. A community member stated that the facilitator was ‘very good at his job of engaging and facilitating, so much so I felt I was directly engaged’. One health knowledge expert commented that ‘the facilitator was great in prompting/facilitating the discussion’.

The benefit of using various tools for co‐design was emphasised by participants in the final evaluation survey. A community member found it useful to use ‘different ways of presenting information … (visual, aural, factual, emotional)’ to accommodate ‘different processing abilities’ of participants. Two health knowledge experts commented that the inclusion of example videos, with various presentation styles from TikTok to longer video explanations, was useful in the co‐design process. Similarly, it was ‘useful to have the answers summarised [throughout the discussion] to give more time to consider these’, as we added responses to a virtual whiteboard throughout the discussion. Health knowledge experts commented that the chat box was an ‘efficient way to share our thoughts’ and to ‘add extra ideas when the conversation was busy’.

There were conflicting comments on the use of polls within Zoom, with three participants (two health knowledge experts and one community member) stating that the polls were useful, while three participants (health knowledge experts) reported technical difficulties with the poll function. This was noted during the sessions, and polls were not used after the first three focus groups for this reason. Instead, where we wanted to identify consensus, the chat function was the most effective way to share opinions from each participant, which were subsequently discussed as a group. There were other technical issues that disrupted the discussion at times, including when participants could not connect their audio, and issues with internet connectivity.

#### 3.3.5 Contribution to Focus Groups

Verbal and written chat contributions to the seven pre‐design and generative focus groups are summarised in Table 1 (for each focus group by cohort) and Table 2 (overall for each cohort). All participants contributed at least once (verbal or chat) in each focus group they attended. There was variation in the number of contributions at each focus group, with the fewest contributions at the first focus group for both cohorts. In total, community members preferred verbal contributions (55% verbal and 45% chat) compared to health knowledge experts, who had a greater proportion of chat contributions (45% verbal and 55% chat). Overall, community members spoke for a median of 7 s longer (*p* = 0.014), but spoke fewer times per participant than health knowledge experts (*p* = 0.003). Health knowledge experts contributed more chats per participant (*p* < 0.001), and the chats were a median of 50 characters longer (*p* < 0.001) compared to community members.

**Table 1:** Participant engagement measured by verbal and chat contributions during each Focus Group 1–7 by cohort.

	Health knowledge experts							Community members						
	Focus group							Focus group						
Participants	1 *N* = 9	2 *N* = 9	3 *N* = 7	4 *N* = 7	5 *N* = 4	6 *N* = 7	7 *N* = 8	1 *N* = 10	2 *N* = 9	3 *N* = 8	4 *N* = 10	5 *N* = 9	6 *N* = 10	7 *N* = 11
Total contributions	45	100	82	79	51	90	74	31	59	74	52	61	55	58
Count of verbal contributions, *n* (%)	29 (64)	37 (37)	35 (43)	35 (44)	27 (53)	40 (44)	30 (41)	23 (74)	30 (51)	31 (42)	32 (62)	39 (64)	32 (58)	29 (50)
Count of chat contributions, *n* (%)	16 (36)	63 (63)	47 (57)	44 (56)	24 (47)	50 (56)	44 (59)	8 (26)	29 (49)	43 (58)	20 (38)	22 (36)	23 (42)	29 (50)
Verbal contributions														
Median contributions per participant (Q1, Q3)	3 (2, 4)	4 (3, 6)	5 (2, 7)	5 (4, 7)	9 (5, 9)	5 (4, 9)	4 (3, 5)	3 (2, 3)	3 (3, 5)	3 (3, 6)	3 (2, 4)	4 (3, 5)	4 (2, 5)	3 (2, 3)
Median duration (seconds; Q1, Q3)	30 (22, 41)	29 (23, 46)	27 (22, 45)	27 (12, 42)	36 (29, 83)	35 (19, 46)	35 (21, 49)	41 (31, 53)	38 (25, 52)	39 (27, 52)	27 (18, 50)	46 (32, 57)	44 (30, 55)	28 (22, 42)
Chat contributions														
Median contributions per participant (Q1, Q3)	2 (1, 2)	5 (5, 10)	8 (2, 12)	6 (2, 12)	6 (3, 10)	7 (3, 11)	6 (3, 8)	0 (0, 1)	3 (0, 5)	5 (3, 7)	2 (0, 4)	2 (0, 4)	2 (0, 4)	3 (0, 5)
Median characters (Q1, Q3)	165 (97, 215)	65 (21, 130)	67 (36, 116)	107 (47, 181)	114 (55, 198)	88 (49, 148)	64 (36, 110)	61 (17, 99)	26 (11, 49)	14 (1, 36)	31 (15, 73)	53 (22, 113)	81 (30, 155)	59 (18, 79)

*Note:* Q1: 25th percentile, Q3: 75th percentile.

**Table 2:** Overall participant engagement by cohort during Focus Groups 1‐7.

	Health knowledge experts *N* = 51	Community members *N* = 67	*p* value
Total contributions	521	390	0.001
Count of verbal contributions, *n* (%)	233 (45)	216 (55)	
Count of chat contributions, *n* (%)	288 (55)	174 (45)	
Verbal			
Median contributions per participant per focus group (Q1, Q3)	4 (3, 6)	3 (2, 5)	0.003
Median duration (seconds; Q1, Q3)	31 (21, 47)	38 (24, 53)	0.014
Chat			
Median contributions per participant per focus group (Q1, Q3)	5 (2, 8)	2 (0, 4)	< 0.001
Median character length (Q1, Q3)	82 (43, 146)	32 (12, 78)	< 0.001

*Note:* Q1: 25th percentile, Q3: 75th percentile.

### 3.4 Participant Satisfaction

Of the 15 respondents to the final evaluation survey from both cohorts, all agreed that they enjoyed being part of the discussions, felt heard and believed their opinions were respected and valued (Figure 3). All health knowledge expert respondents (*n* = 6) believed their contributions influenced the content of the digital platform, while 66% (*n* = 6) of the community members agreed. Overall, the health knowledge experts reported that it was a ‘great experience’ and was ‘engaging and interesting’. Feedback from community members aligned, with several perceiving the focus groups as ‘enjoyable’ and an ‘excellent experience’. All respondents agreed they would be willing to be part of a similar project again.

A common theme was the impact that co‐design had on the respondents. A health knowledge expert reported they ‘learnt a lot’ and another highlighted the opportunity to contribute to ‘such an importance [sic] project and liaise with other professionals working in the same clinical area’. Community members agreed, with one respondent commenting on the importance of being ‘involved in a program which may be so beneficial in preventing strokes’, and another that their ‘beliefs were validated’. One community member stated that the impact of their involvement meant their ‘stroke has a positive outcome’ and another commented that the process ‘made me think about my own stroke’ and they ‘had to think about the things under discussion in a different way’.

Notably, several (*n* = 4) health knowledge experts commented on the opportunity of co‐designing with a ‘diverse range of professionals’ who had ‘different clinical background and experiences’. And one reported they were ‘inspired … to think and explore more about stroke education on both patients and staff’. This was echoed among community members, with one respondent stating that through the co‐design process they were ‘exposed to a variety of lived experiences, which were different, but ultimately all the same’. One community member commented that ‘I felt we all knew what was the most important thing to say, we just went about it in different ways’.

The final evaluative focus group was described as very important by the participants. One health knowledge expert attended the final focus group and commented that ‘it's nice to see the end product’ and that they ‘really value this kind of follow up session’. Similar comments were made by the community members, who stated that ‘[evaluation] is a really good section of the process’ and that the final product ‘incorporated a lot of feedback raised from the general public perspective and the user side’. One community member commented that ‘you have always valued our opinions, you have always listened to what we had to say,’ and this had been ‘reflected in … the outcome of the project’.

## 4 Discussion

The interrelated attributes of coordination, participant engagement and satisfaction contributed to the success of our co‐design project. The focus groups were reported to be well organised, structured and facilitated by experts in co‐design, and this level of coordination likely contributed to participants' sustained engagement and satisfaction reported throughout the process. Participants also identified benefits to their participation and enjoyed being involved, which also likely contributed to their level of engagement. Overall, the following three components were critical to the success of this collaborative co‐design process: coordination, participant engagement and participant satisfaction.

Certain elements of co‐design research, such as the timeframe, method of co‐design (e.g., focus group or workshop) and the number and type of participants involved, are commonly reported [24]. While there is no consistent way to report co‐design methodology, there is growing interest in establishing more consistent and transparent evaluation practices, as reflected in recently published frameworks and protocols for guideline development [11, 25]. Objective evaluation of the co‐design process is often lacking as it is difficult to measure aspects of participation (e.g., capacity building and shared power) [4]. There are numerous frameworks to evaluate consumer involvement in research, but these are often poorly used beyond the authors who developed them [26]. Given the differences in co‐design approaches, a universal method to evaluate co‐design is not necessarily the solution. Instead, it may be more useful and relevant to evaluate each co‐design project using a purpose‐built framework based on elements of existing work [26]. For that reason, we evaluated our co‐design approach using relevant concepts from the Co‐Design Evaluation Framework [11] and included broad themes of partnership described in other co‐design literature (project management, communication, reimbursement and collaborative learning) [26], which may guide and strengthen other co‐design projects.

There was considerable interest in participation in this study from people with diverse perspectives. However, we were unable to recruit medical practitioners, which is a common limitation of similar co‐design studies [27]. We offered equal reimbursement to both cohorts [28], which they found satisfactory, but some guidelines suggest that reimbursement for participants acting in their professional capacity should be in line with industry pay rates [29]. It was also difficult to recruit people in the community without personal experience of stroke. Stroke prevention is of particular interest to people with lived experience of stroke, and as a result, the community member cohort was predominantly people with lived experience and people already involved in the Stroke Foundation StrokeSafe program [14]. This may explain why participant engagement and retention were so high in the community member cohort. We acknowledge that a different group of participants with no interest in stroke may have had different perspectives.

As Love Your Brain is a digital platform, online focus groups were the ideal medium to ensure we engaged relevant stakeholders. Compared to in‐person focus groups, video conferencing is cost‐effective, saves time and allows participants to join from anywhere they feel comfortable (including one participant who joined from the side of a highway whilst on holiday) [30, 31]. Our experience aligns with similar co‐design projects, where remote focus groups resulted in more sustainable involvement as participants could join as it suited them, in addition to cost and time savings [32]. As researchers, the ability to rewatch the focus group recordings ensured that we incorporated participant suggestions and feedback into the digital platform and had a detailed and accurate record of each session. There are inherent limitations to video conferencing, including restricting access to people with appropriate technology and digital literacy [33]. Technical issues disrupted the discussion at times, similar to other virtual research projects [30, 31, 34, 35]. While these challenges were difficult to troubleshoot during the focus groups, they fostered collaboration between participants and the research team, who helped each other to resolve issues.

While conducting 16 focus groups allowed time to follow an iterative co‐design process, this large number of focus groups took longer than expected and required a significant investment of research staff time. Our estimate of the time invested is likely to be conservative, as we did not include time to transcribe focus groups and analyse and synthesise data or for activities to support the broader project, such as funding acquisition, protocol development, ethics approval, committee oversight, risk management and budgeting. These activities were excluded because they do not directly influence participant experience and engagement in the focus groups. Other researchers have identified that co‐design requires adequate resources to maximise the process [36]. Participants in our study valued the benefits of a structured and well‐organised co‐design process, justifying the time invested by research staff. Our iterative co‐design process allowed us to incorporate participant feedback to refine our processes and develop ideas across multiple focus groups, which ultimately enhanced the co‐design experience. To optimise attendance at multiple focus groups, we recommend scheduling all dates and times well in advance, as suggested by investigators in an Austrian co‐design project [37].

Participants were actively engaged, and it is likely that the high level of participant engagement [38] resulted in excellent retention. Having the same expert facilitators deliver each focus group helped to develop a strong rapport between the participants and facilitators. Zechmeister‐Koss et al. [37] described the importance of building relationships between co‐design participants for successful project outcomes. For both cohorts in our study, the first focus group had fewer contributions, which was largely attributable to the greater proportion of time required to introduce the project, the other participants and the co‐design process. After the first focus group, we observed that participants, particularly community members, appeared to be more familiar with the other participants, the structure of the sessions and Zoom functionality.

Participants were committed to this project and were eager to contribute. All participants actively contributed in each focus group they attended, and no single person dominated the conversations. It is important to tailor co‐design activities to different stakeholders [4], and our findings suggest that preferences differed between cohorts. There was considerable use of the chat function, particularly by health knowledge experts, demonstrating the benefit of having both verbal and written options to contribute. The chat function provided the opportunity for participants to contribute ideas after the discussion had moved on, thereby not interrupting the flow of the focus group. It was also useful having a virtual whiteboard to summarise the discussion in real‐time, and this also encouraged participants to build on or disagree with the ideas of others. We were surprised that participants wanted pre‐reading material, as we thought this would be an additional burden on them. Throughout the co‐design process, the pre‐reading materials were reviewed thoroughly by participants and prompted unsolicited responses from participants ahead of the focus groups. Community members felt the pre‐reading was more useful than the health knowledge experts, but this may be due to the different levels of expertise, project expectations and information needs. Active engagement is associated with shared power between stakeholders and valuing people's ideas [39]. We found that separate focus groups for health knowledge experts and community members helped to overcome potential inherent power imbalance between these stakeholders [40] and allowed tailored approaches, which may have increased the level of meaningful engagement within each cohort.

A critical step in the co‐design process was the final evaluative focus group, where participants previewed the co‐designed digital platform. Notably, there were fewer participants in the final evaluative focus group, potentially due to the time gap and as we were no longer asking for knowledge input. Those who attended witnessed how their suggestions and feedback had been incorporated into the final product, which confirmed that their contribution to co‐design was heard, worthwhile and valuable. While the final focus group was highly regarded by participants in our study, an international survey of co‐design researchers reported that 42% of respondents skipped a final ‘celebration/review event’ [41]. We recommend that other co‐design projects include a final evaluative focus group, given how important it was for the participants.

### 4.1 Strengths and Limitations

Our distributed co‐design method, where health knowledge experts and community members were engaged separately, allowed us to balance potential inequities between stakeholder groups and achieve equitable contributions from participants. In contrast, other investigators have conducted successful co‐design with combined groups where participants valued the learnings and partnerships formed between health professionals and people with lived experience [42].

The project investigators who attended the focus groups may have affected the outcomes. Participants may have been restricted in freely expressing their views in the presence of the project investigators [18]. Alternatively, by attending the focus groups, the investigators demonstrated their commitment to the project and interest in the participants' contributions, which may have encouraged participation [43].

Proficiency in the English language may have excluded people from culturally and linguistically diverse backgrounds from participating. Co‐designing a solution with these populations may help overcome known barriers to using technology for health, including language, digital literacy and accessibility [44]. After our initial effectiveness trial, we plan to further develop and tailor the Love Your Brain digital platform content and delivery with, and for, cultural and language diverse groups.

Measuring participant engagement by quantifying contributions at focus groups is not a validated measure, but it provides an indication of participation. We acknowledge that shorter or less frequent contributions from participants can also provide meaningful insight. Authors of this manuscript were directly involved in the co‐design process and overarching research project; therefore, this is not an independent evaluation. Only a 2‐week period was evaluated to determine the level of resourcing required to conduct focus groups, with additional administration (such as recruitment of staff, onboarding, annual leave and steering committee) not considered.

### 4.2 Future Suggestions

There may be benefits of smaller focus groups. We prioritised diversity with up to 15 participants in each cohort, but recognise that smaller groups may have provided more in‐depth conversation. However, there is a balance, as the focus groups we conducted with four or fewer participants were more intense for the participants who had to constantly contribute to the discussion, with fewer comments to build upon. Other authors suggest that interpersonal dynamics are more important than focus group size [45], and small groups may be preferable in health research to give people time to tell their story [46].

We did not use breakout rooms due to the increased amount of coordination and to reduce the time involvement of participants. We preferred that all participants hear and contribute to the same discussion, rather than having separate discussions facilitated by different researchers, and then spend time summarising the small group discussions. We acknowledge that this method may be effective for enhancing small group collaboration, but it introduces additional resourcing and technological requirements [34].

## 5 Conclusion

We completed a large co‐design project, with seven pre‐design and generative focus groups, and one evaluative focus group per cohort. We provide a novel quantitative method for assessing the level of engagement, using the count and duration/length of contributions captured using technology. This metric enabled comparison between cohorts to identify their preferred method of communication in focus groups (e.g., community members preferred verbal contributions, while health knowledge experts preferred to write messages though the chat function), and this approach should be used to enhance future research by offering a range of appropriate communication options for the participants involved.

The engagement of key stakeholders over an extended period enabled the development of familiarity and rapport between facilitators and the participants, which benefited the iterative design of a final product that the participants took pride in creating. For successful co‐design, we recommend consideration and investment in the underpinning processes of recruitment, coordination, participant engagement and satisfaction. We advocate for co‐design to involve repeated meetings with the same stakeholders. We identified that re‐engagement provided familiarity with the structure, facilitators and other participants, which likely enhanced engagement, discussion and satisfaction with the co‐design process for all participants.

## Author Contributions

**Catherine Burns:** writing – original draft, formal analysis, methodology, investigation. **Monique F. Kilkenny:** funding acquisition, writing – review and editing, methodology, supervision, investigation. **Tara Purvis:** funding acquisition, writing – review and editing, methodology, supervision, investigation. **Seana L. Gall:** funding acquisition, writing – review and editing, investigation. **Christine Farmer:** writing – review and editing, investigation. **Seamus Barker:** writing – review and editing, investigation. **Brenda Booth:** writing – review and editing, funding acquisition. **Janet E. Bray:** funding acquisition, writing – review and editing. **Dominique A. Cadilhac:** funding acquisition, writing – review and editing. **Jan Cameron:** funding acquisition, writing – review and editing. **Lachlan L. Dalli:** writing – review and editing. **Stephanie Ho:** writing – review and editing. **Eleanor Horton:** funding acquisition, writing – review and editing. **Timothy Kleinig:** funding acquisition, writing – review and editing. **Lisa Murphy:** funding acquisition, writing – review and editing. **Mark R. Nelson:** funding acquisition, writing – review and editing. **Muideen T. Olaiya:** funding acquisition, writing – review and editing. **Amanda G. Thrift:** funding acquisition, writing – review and editing. **Rosanne Freak‐Poli:** writing – review and editing, conceptualisation, methodology, supervision, investigation.

## Ethics Statement

Ethical approval for this study was received from the Monash University Human Research and Ethics Committee (#35899).

## Conflicts of Interest

M.F.K. and D.A.C. are members of the Australian Stroke Clinical Registry Management Committee; A.G.T. is a previous Board Member of Stroke Foundation; M.F.K. is a member of the Research Advisory Committee of Stroke Foundation; S.L.G. is Chair of Stroke Foundation's Stroke Prevention Advisory Committee and a member of their Clinical Council. M.R.N. reports membership of a Novartis lipids advisory board outside the submitted work. All other authors report no conflicts.

## Supporting information


**Supporting Table I.** Distribution of Expression of Interest Form. **Supporting Table II.** Expression of Interest Form. **Supporting Table III.** Post‐Session Feedback Survey. **Supporting Table IV.** Final Evaluation Survey. **Supporting Table V.** Participant Characteristics. **Supporting Figure I.** Example of Pre‐Reading Material.

## Data Availability

The data that support the findings of this study are available from the corresponding author (R.F.) upon reasonable request.
