# Supplementary material for: Processes Underpinning Successful Co‐Design: Lessons From a Digital Health Project
Source: Health Expect. 2025 May 22;28(3):e70272. doi: 10.1111/hex.70272 (PMC12098295; doi:10.1111/hex.70272)
Supplement: Supplementary file 1 — Supporting Table I. Distribution of Expression of Interest Form. Supporting Table II. Expression of Interest Form. Supporting Table III. Post‐Session Feedback Survey. Supporting Table IV. Final Evaluation Survey. Supporting Table V. Participant Characteristics. Supporting Figure I. Example of Pre‐Reading Material. [file HEX-28-e70272-s001.docx]

# Supplemental Material

# Processes Underpinning Successful Co-design: Lessons from a Digital Health Project

Catherine Burns, Monique F. Kilkenny, Tara Purvis, Seana L. Gall, Christine Farmer^3^, Seamus Barker, Brenda Booth, Janet E. Bray, Dominique A. Cadilhac, Jan Cameron, Lachlan L. Dalli, Stephanie Ho, Eleanor Horton, Timothy Kleinig, Lisa Murphy, Mark R. Nelson, Muideen T. Olaiya, Amanda G. Thrift, Rosanne Freak-Poli

# Supplementary Tables

**Supplementary Table I.** Distribution of Expression of Interest Form

**Supplementary Table II.** Expression of Interest Form

**Supplementary Table III.** Post-Session Feedback Survey

**Supplementary Table IV.** Final Evaluation Survey

**Supplementary Table V.** Participant Characteristics

# Supplementary Figures

**Supplementary Figure I.** Example of Pre-Reading Material

# Supplementary Table I. Distribution of Expression of Interest form

| Date | Action |
| --- | --- |
| 18 April 2023 | - Email invitation and flyer sent to co-investigators for circulation. - Recruitment email forwarded to Stroke Foundation StrokeSafe speakers (https://strokefoundation.org.au/what-we-do/prevention-programs/strokesafe-speakers). |
| 19 April 2023 | - Recruitment email forwarded to Community Reference Group at Menzies Institute for Medical Research, to individuals with lived experience who participated in previous stroke research projects, and Stroke Foundation Consumer Council members. |
| 27 April 2023 | - Recruitment email forwarded to research collaborators at the Florey Institute, Queensland Aphasia Research Centre, Centre of Cardiovascular Research & Education in Therapeutics, and Victorian Heart Institute. |
| 28 April 2023 | - Reminder email sent to co-investigators to seek support recruiting health knowledge experts. - Recruitment email forwarded to clinician researcher collaborators (nurse, stroke neurologist, physiotherapist, occupational therapist). |
| 1 May 2023 | - Recruitment email forwarded to New South Wales Agency for Clinical Innovation, and Stroke Foundation employees. |
| 2 May 2023 | - Recruitment email forwarded to Stroke and Ageing Group at Monash University, and Stroke Society of Australasia. - Recruitment email forwarded to Heart Foundation for inclusion in the monthly consumer newsletter. |
| 3 May 2023 | - Recruitment email forwarded to Florey Institute for inclusion in newsletters to communities of practice and contributors. |
| 2-4 May 2023 | - Hard-copy recruitment flyers were available at an exhibition stand at the Preventative Health Conference 2023, Adelaide. |

# Supplementary Table II. Expression of Interest form

| Demographic Questions | | |
| --- | --- | --- |
| Question | **Response Options** | **Field Attributes** |
| I would like to participate as | 1. Community member  2. Health knowledge expert | Radio button, required |
| Full name |  | Free text, required |
| Age | 1. 17 and under  2. 18-24  3. 25-34  4. 35-44  5. 45-54  6. 55-64  7. 65-74  8. 75 and over | Dropdown, required |
| What is your gender? | 1. Woman  2. Man  3. Non-binary/gender diverse  4. Prefer not to say | Checkboxes, required |
| Where do you live? | 1. Australian Capital Territory  2. New South Wales  3. Northern Territory  4. Queensland  5. South Australia  6. Tasmania  7. Victoria  8. Western Australia | Dropdown, required |
| What is your postcode? |  | Free text, required |
| Email address |  | Free text, required |
| Which of these statements best describes you? | 1. Person with lived experience of stroke  2. Caregiver for a person with lived experience of stroke  3. Member of the public who is interested in preventing stroke  4. Member of the public who has/had a relative or friend affected by stroke  5. Member of the public who has/had a work colleague affected by stroke  6. Healthcare professional treating stroke patients  7. Healthcare professional treating people at risk of stroke  8. Researcher who is interested in preventing stroke  9. Other, please specify  10. Prefer not to say | Checkboxes, required |

| Health knowledge experts only | |  |
| --- | --- | --- |
| Question | **Response Options** | **Field Attributes** |
| What is your profession? | 1. Clinician  2. Researcher  3. Clinician researcher  4. Representative of health-related charity  5. Other, please specify | Radio button, required |
| What is your job title? |  | Free text, required |
| What are your qualifications? |  | Free text, required |
| Years of experience | 1. 1-4 years  2. 5-9 years  3. 10-14 years  4. 15 and over years | Dropdown, required |

| Community members only | |  |
| --- | --- | --- |
| Question | **Response Options** | **Field Attributes** |
| Telephone number |  | Free text, required |
| Which cultural or ethnic group do you identify with? | 1. Australian  2. Australian Aboriginal  3. Australian South Sea Islander  4. Torres Strait Islander  5. Norfolk Islander  6. Māori  7. New Zealander  8. British  9. Irish  10. Western European  11. Northern European  12. Southern European  13. South Eastern European  14. Eastern European  15. Arab  16. Jewish  17. Peoples of the Sudan  18. Other North African and Middle Eastern  19. Mainland South-East Asian  20. Maritime South-East Asian  21. Chinese Asian  22. Other North-East Asian  23. Southern Asian  24. Central Asian  25. North American  26. Central American  27. Caribbean Islander  28. Central and West African  29. Southern and East African | Dropdown, required |
| Highest level of education | 1. Less than Year 12 equivalent  2. Year 12 equivalent  3. Certificate/diploma  4. Bachelor degree  5. Postgraduate degree | Dropdown, required |
| Do you have cardiovascular disease? This includes conditions that affect your heart or circulation such as heart disease, stroke or peripheral arterial disease | 1. Yes  2. No | Radio button, required |
| *If yes*, select all relevant conditions | 1. Stroke or transient ischaemic attack (TIA)  2. Coronary heart disease (including heart attack and angina)  3. Congenital heart disease  4. Rheumatic heart disease  5. Heart failure  6. Peripheral arterial disease  7. Deep vein thrombosis or pulmonary embolism  8. Other, please specify | Checkboxes, required |
| Do you have any of the following risk factors? | 1. High blood pressure  2. High cholesterol  3. Type 2 diabetes (diabetes mellitus)  4. Current smoker  5. High alcohol consumption  6. Obesity  7. Previous stroke or transient ischaemic attack (TIA)  8. None of the above | Checkboxes, required |

# Supplementary Table III. Post-Session Feedback Survey

Survey questions after Focus Groups 1-6:

| Question | Field Attributes |
| --- | --- |
| Reflecting on the discussion sessions, was there anything that you didn’t have the opportunity to say, or want to add? | Free text |
| Was there any additional information that might have been helpful to receive prior to the discussion sessions? | Free text |
| Do you have any suggestions to improve the experience of people who are contributing to the discussion? | Free text |
| Please let us know if you have any other general comments about the discussion sessions | Free text |

Additional questions after Focus Group 1:

| Question | Response Options | Field Attributes |
| --- | --- | --- |
| Did the time suit you? | 1. Yes  2. No | Radio button, required |
| If no, please suggest alternative time |  | Free text |
| Did the day of the week suit you? | 1. Yes  2. No | Radio button, required |
| If no, please suggest alternative day |  | Free text |

Additional questions after Focus Group 1 and/or 2:

| How much do you agree or disagree with the following statements regarding the Love Your Brain project discussion session? | | | |
| --- | --- | --- | --- |
| Focus Group | **Statement** | **Response Options** | **Field Attributes** |
| 1 | I had no problems connecting and participating via Zoom | 1. Strongly agree  2. Agree  3. Neither agree nor disagree  4. Disagree  5. Strongly disagree | Radio button, required |
| 1 | The technology was easy to use |  |  |
| 1 | It was clear what was expected of me |  |  |
| 1,2 | I was able to interact with the facilitators |  |  |
| 1 | I was able to interact with other participants |  |  |
| 1,2 | I could follow what was happening during the discussion sessions |  |  |
| 1,2 | I felt I was able to express my views |  |  |
| 1,2 | The discussion gave all participants a chance to contribute their thoughts |  |  |
| 1,2 | The length of the discussion sessions was appropriate |  |  |
| 1,2 | The size of the discussion group was appropriate |  |  |
| 1 | I would have preferred that the group numbers were smaller |  |  |
| 2 | The Zoom polls were useful to express my opinion |  |  |
| 2 | It was easy to use the Zoom polls |  |  |
| 2 | I would like more Zoom polls |  |  |

# Supplementary Table IV. Final Evaluation Survey

Survey questions after Focus Group 7

| Please select one response for each statement | | |
| --- | --- | --- |
| Statement | **Response Options** | **Field Attributes** |
| I have enjoyed being part of the discussions | 1. Strongly agree  2. Agree  3. Neutral  4. Disagree  5. Strongly disagree | Radio button |
| I felt like I was heard during the discussions |  |  |
| I believe my opinions were respected and valued |  |  |
| I believe that what I shared will influence the content and delivery of the digital platform |  |  |
| I feel that I was fairly reimbursed for my time and input |  |  |
| The pre-reading was useful and engaging |  |  |
| The discussion questions were framed at an appropriate level for me |  |  |
| The discussion was well organised and timely |  |  |
| The facilitator had adequate knowledge of the topic |  |  |
| I would be willing to be part of a similar project in the future to help improve stroke prevention |  |  |

| Question | Field Attributes |
| --- | --- |
| How would you describe your overall experience being involved in the co-design discussions? | Free text |
| In what ways did being involved in the co-design discussions have an impact on you? | Free text |
| What activities were useful in the discussions? | Free text |
| What activities were not useful in the discussions? | Free text |
| What could be done to improve/assist your participation in future? | Free text |
| Do you have any further comments on the digital platform (MOOC and text messages)? | Free text |

MOOC: massive open online course

# Supplementary Table V. Participant Characteristics

|  | **Health Knowledge Experts** | | **Community Members** | | |
| --- | --- | --- | --- | --- | --- |
|  | **Invited***  **N=16**  **n (%)** | **Participated**  **N=10**  **n (%)** | **EOI**  **N=28**  **n (%)** | **Invited**  **N=20**  **n (%)** | **Participated**  **N=12**  **n (%)** |
| **Demographics** |  |  |  |  |  |
| <65 years | 16 (100) | 10 (100) | 16 (57) | 10 (50) | 7 (58) |
| Woman | 14 (88) | 8 (80) | 22 (79) | 14 (70) | 7 (58) |
| **Location** |  |  |  |  |  |
| New South Wales | 11 (69) | 6 (60) | 6 (21) | 3 (15) | 3 (25) |
| Victoria | 5 (31) | 4 (40) | 6 (21) | 4 (20) | 2 (17) |
| Queensland | 0 (0) | 0 (0) | 3 (11) | 3 (15) | 2 (17) |
| Western Australia | 0 (0) | 0 (0) | 2 (7) | 2 (10) | 0 (0) |
| South Australia | 0 (0) | 0 (0) | 1 (4) | 1 (5) | 1 (8) |
| Tasmania | 0 (0) | 0 (0) | 8 (29) | 5 (25) | 3 (25) |
| Australian Capital Territory | 0 (0) | 0 (0) | 2 (7) | 2 (10) | 1 (8) |
| Northern Territory | 0 (0) | 0 (0) | 0 (0) | 0 (0) | 0 (0) |
| **Highest level of education** |  |  |  |  |  |
| Less than Year 12 equivalent | 0 (0) | 0 (0) | 0 (0) | 0 (0) | 0 (0) |
| Year 12 equivalent | 0 (0) | 0 (0) | 1 (4) | 1 (5) | 0 (0) |
| Certificate/Diploma | 0 (0) | 0 (0) | 6 (21) | 4 (20) | 3 (25) |
| Bachelor degree | 5 (31) | 2 (20) | 12 (43) | 9 (45) | 6 (50) |
| Postgraduate degree | 11 (69) | 8 (80) | 9 (32) | 6 (30) | 3 (25) |
| **Profession** |  |  |  |  |  |
| Clinician | 10 (63) | 5 (50) | - | - | - |
| Researcher | 5 (31) | 5 (50) | - | - | - |
| Clinician Researcher | 1 (6) | 0 (0) | - | - | - |
| **Years of Experience** |  |  |  |  |  |
| 1-4 years | 1 (6) | 1 (10) | - | - | - |
| 5-9 years | 3 (19) | 3 (30) | - | - | - |
| 10-14 years | 4 (25) | 3 (30) | - | - | - |
| 15+ years | 6 (38) | 3 (30) | - | - | - |

EOI: expression of interest.
*All health knowledge experts who expressed interest in participating were invited.

# Supplementary Figure I. Example of Pre-Reading Material
